# HOsT: Towards a Low-Cost Fog Solution via Smart Objects to Deal with the Heterogeneity of Data in a Residential Environment

**DOI:** 10.3390/s22166257

**Published:** 2022-08-20

**Authors:** Geraldo P. Rocha Filho, Artur H. Brandão, Renato A. Nobre, Rodolfo I. Meneguette, Heitor Freitas, Vinícius P. Gonçalves

**Affiliations:** 1 Department of Computer Science, University of Brasília, Brasília 70910-900, Brazil; 2 Computer Science Department, Università degli Studi di Milano, 20122 Milano, Italy; 3 Institute of Mathematical and Computer Sciences, University of São Paulo, São Carlos 13560-970, Brazil; 4 Sidia R&D Institute, Manaus 69055-035, Brazil; 5 Electrical Engineering Department, University of Brasília, Brasília 70910-900, Brazil

**Keywords:** fog computing, IoT, MQTT, smart objects, computational resources, data heterogeneity, interoperability

## Abstract

With the fast and unstoppable development of technology, the amount of available technological devices and the data they produce is overwhelming. In analyzing the context of a smart home, a diverse group of intelligent devices generating constant reports of its environment information is needed for the proper control of the house. Due to this demand, many possible solutions have been developed in the literature to assess the need for processing power and storage capacity. This work proposes HOsT (home-context-aware fog-computing solution)—a solution that addresses the problems of data heterogeneity and the interoperability of smart objects in the context of a smart home. HOsT was modeled to compose a set of intelligent objects to form a computational infrastructure in fog. A publish/subscribe communication module was implemented to abstract the details of communication between objects to disseminate heterogeneous information. A performance evaluation was carried out to validate HOsT. The results show evidence of efficiency in the communication infrastructure; and in the impact of HOsT compared with a cloud infrastructure. Furthermore, HOsT provides scalability about the number of devices acting simultaneously and demonstrates its ability to work with different devices.

## 1. Introduction

In recent years, information has become one of the most valuable assets globally [1]. Such information is obtained from raw data, documented observations, or measurement results [2]. There are various ways of obtaining data, one of them is through smart objects. Those objects can: (i) determine the value of a local event; (ii) identify events of interest; (iii) process such events on and off the network; (iv) detect objects of interest; and (v) establish data communication and scalability.

With advancing technology in the field of microelectromechanical systems, there has been significant growth in the use of smart objects within the residential context [3,4,5,6], such as smart TVs, smartwatches, and smart refrigerators. Furthermore, with these objects connecting all the physical assets and communication itself through the internet, it is a revolutionary concept from IoT (Internet of Things) [7]. In this regard, smart homes are emerging as promising sources of information and arouse interest among researchers for proposing models and applications to perform smart decision making [8,9]. A smart home can be defined as a set of smart objects contained in a household to automate daily tasks and keep track of data provided by the environment to the user, to manage the user’s residential resources [10].

It is noteworthy that due to a shortage of computational resources contained in the smart objects, developing—in the residential setting—services that use intercommunication between objects is not a trivial task. Solving this limitation becomes even more challenging due to data heterogeneity and interoperability among the objects present in the home. As an example of this challenge, all the data collected by the temperature and the humidity sensors of Alexa devices are received by the Alexa application and the AR conditional. Later, both applications obtain the requested data and perform some pre-established actions. For example, the AR conditional temperature lowers as the environmental temperature increases. Additionally, Alexa will provide an alert if the humidity reaches below twenty percent. To deal with such challenges, a promising path is the use of fog computing [11] along with the communication paradigm publish/subscribe (Pub/Sub) [12]. Fog computing is a highly virtualized platform that provides processing, storage, and networking services between end devices and traditional cloud servers—that is, tasks are performed cooperatively among the smart objects contained within an environment through communication [11]. The Pub/Sub paradigm allows reliable asynchronous communication among objects, from multiple event sources to their respective groups of interests [13].

In this context, fog computing is characterized as an environment with many decentralized and heterogeneous objects that can cooperate with each other. This cooperation takes place through the use of the local internet, which improves storage performance and task processes without the interference of third parties, such as the use of the cloud [14]. Thus, all objects that are contained in this fog environment are called fog nodes—that is, they are devices that bring processing, storage, and internet services to the edge of the internet. They are also characterized as purpose devices [15]. To have a communication between these objects belonging to the same fog environment, the Pub/Sub paradigm is used that is composed of objects that contain characteristics of publishers or subscribers that send and receive messages, respectively, in an uncoupled and asynchronous manner [13].

Different approaches have been proposed to deal with the issue of data heterogeneity and interoperability among devices in the same environment [16,17,18,19,20]. Some approaches [16,18] use MQTT (message queue telemetry transport) to create clusters for scalability in IoT devices, and others to provide services in IoT applications. However, such approaches use MQTT only to provide specific services. Other approaches [19,20] use the fog computing paradigm to take advantage of residential devices’ local processing to reduce the processing time in the decision-making process. However, the approaches focus only on applications, not worrying about the increase in smart objects.

Despite significant efforts in this area, investigating the interoperability of communication between devices, taking advantage of contents implicitly disseminated in a residential context based on fog computing is an open field of research that this work investigates. Because of this, the following research question was formulated to guide this work: Is the use of fog computing, as an infrastructure, combined with Pub/Sub, as a communication module, capable of improving the computational costs of a residential environment? Thus, this work is based on the hypothesis that fog computing combined with Pub/Sub can provide scalability to deal with data heterogeneity within the residential context and optimize its services.

Therefore, this paper presents HOsT (home-context-aware fog-computing solution), a solution to the problems of data heterogeneity and interoperability of smart objects in the context of the smart home. For this purpose, a fog-computing environment was set up through household smart objects to build the HOsT infrastructure. To disseminate information, a Pub/Sub communication module was implemented in the infrastructure with the aim of improving the computational and operational costs originating from the variation of the quantity of fog nodes and the amount of data exchanged among the objects. The results show that HOsT performs satisfactorily in relation to the devices computational resources and the proposed solution infrastructure.

The remainder of this paper is organized as follows. Section 2 presents some limitations related to other works that, in their majority, worked with the MQTT communication mechanism. Section 3 describes, in detail, how HOsT works and how its components act during the execution process. Its validation is presented in Section 4; meanwhile, Section 5 presents our conclusions and plans for future work.

## 2. Related Works

This section will address smart-environment-related works that contain a similar concept to an MQTT connection for communication among devices. As described in [18], the authors show how efficient it is to use an MQTT broker alongside AWS (Amazon Web Services) to manage a small IoT (Internet of Things) application in a smart home environment. This methodology was proposed through the end-to-end implementation of the suggested scenario. Thus, the authors developed hardware that captures temperature and smoke detection data. This hardware connects to the MQTT created on AWS, and it was developed an application for capturing events transmitted by the MQTT. Therefore, this study demonstrated great ease in developing an MQTT application on AWS for small IoT applications. However, there is a performance limitation regarding the application usage since a likely increase in the number of devices could cause a performance problem on the broker.

In another study [19], a method of handling a large amount of heterogeneous data captured by IoT devices using a cloud storage feature called Apache Kafka is presented. For this purpose, they developed a REST web service interface enabling easy use of the utilized platform and Google Cloud as storage. As a result, the calculated metrics for measuring the performance of the whole message transmission to the cloud were considered efficient, with the possibility, therefore, of using this architecture for real-time data transmission. Finally, the routes for sending data directly to the cloud were analyzed, which may have expanded the study to fog communication among local devices.

Based on [17], the creation of MQTT broker clustering was proposed, which aimed to achieve scalability and low cost through a large number of connected IoT devices in the same environment. For this purpose, a visualization system was created to analyze the cluster data regarding CPU usage, memory, and network traffic. As a way of simulating the proposal, Raspberry Pi devices were employed as nodes, and, through the Mosquitto, the MQTT broker was used for message communication among them, in addition to using docker and NGINX. Furthermore, scalability was acquired through the use of docker containers and, with NGINX implemented at the backend of MQTT brokers, the researchers achieved a positive outcome regarding the assessed metrics found in the visualization analyses. A way of improving the assessment would be to cover the amount of MQTT protocols, such as message exchange, to more fully compare metrics.

With the proposition of managing the applications of a residence through fog computing, [21] suggest a neuro-fog-control system via smart objects. Thus, this solution’s goal is to ensure fast decision making when executing the system locally in the infrastructure designed by the authors. The internal data dissemination was performed through Pub/Sub methods, which made the scale of devices viable without causing communication problems among new devices. Furthermore, on fog computing, [20] propose a low-cost residential automation system to improve the resident decision-making process. To this end, the proposed system aims to learn the applications through a classifier ensemble. Considering the internal settings of the communication protocols employed in the study, there would be a possibility increasing the message size and then analyzing for interference in the evaluated metrics. Therefore, the solution presented in this research differs from others in the communication interface used to address data heterogeneity and interoperability among the devices in a smart home.

In the search for a smart home system at a low and flexible cost, [16] demonstrates the use of an Android application that communicates through a micro-web server to provide numerous interactive features among devices. To this end, Arduino Ethernet was used to connect sensors to capture temperature, humidity, and gas sensitivity from the environment. Thus, such devices communicate with the developed application, and this application’s purpose is to control the sensors and capture the entered data. The application managed to attain the expected result through the tests, demonstrating viability and effectiveness in communication between smart home devices and Android applications. However, since there was only verification of the application connectivity success, other means of communication among the devices could be verified, such as MQTT. With this communication, there could be a higher speed of data transfer and easy maintenance of the proposed system due to the great scalability and operability capacity of its usage compared with other means of communication, as shown in surveys [22].

In [23], a comparison is made between fog and cloud computing usage regarding the data traffic metrics in both environments. Thus, this paper provides data on latency and delays caused by data transfer, which may interfere with the communication performance depending on the environment. Therefore, a significant increase in the fog environment performance is clear through the collected data of both environments, considering the desire for latency and avoiding congestion due to internet traffic. Thus, there is still a need to evaluate communication methods within the fog setup to compare the same metrics.

Table 1 shows the above-mentioned limitations, comparing them with this research. We can observe that the works do not make an aggregation of different technologies, i.e., the work that uses the Pub/Sub paradigm does not use fog computing, and vice versa. Furthermore, studies offer a greater focus on the developed application without considering a possible increase in objects in their projects and how this could affect the proposed approaches. Thus, HOsT uses fog computing and Pub/Sub to provide more efficient intercommunication between heterogeneous devices to meet the needs of the services used.

## 3. HOsT: Home-Context-Aware Fog-Computing Solution

This section presents HOsT, a fog-computing solution to address the problem of data heterogeneity and the interoperability of smart objects within the residential context. HOsT was designed to have a set of smart objects provided in a smart house itself; these objects would be connected to the internet, forming a fog-computing environment. Such an environment, also called edge computing, creates cooperation between the use of the internet and the use of the heterogeneous devices that work together to improve storage performance and task processing for being on the network edge layer [11]. Once the cloud computing confirms its limits [24], fog computing becomes a viable option by delivering low latency and an improvement in response rate among the objects that constitute the environment.

**Figure 1:**
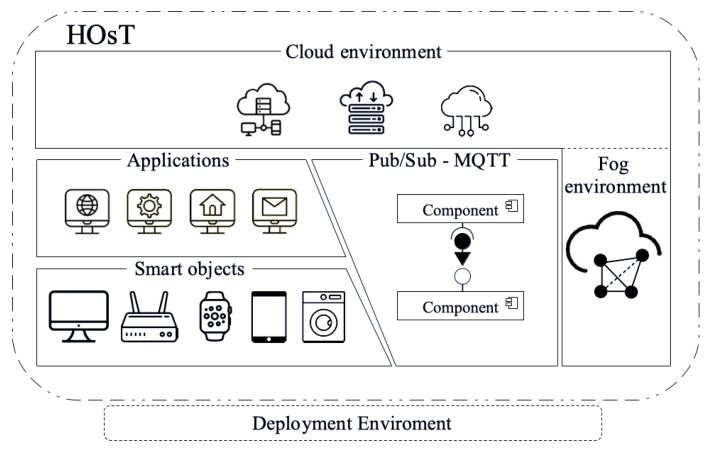
HOsT operating scenario.

A module that abstracts the details of smart objects communication based on the Pub/Sub communication module was implemented on HOsT. Due to the decoupling offered by the final parts through Pub/Sub, it is possible to create interest groups for the collected data. In this regard, it is feasible to ensure that the environment modules, objects, and applications can communicate among themselves, assisting in the data dissemination process as shown in Figure 1.

HOsT contains smart objects that produce data and send them to the environment module through the MQTT–Pub/Sub connection, as shown in Figure 1. Furthermore, the applications work upon the data collected in the environment. To do so, the environment’s operational infrastructure of HOsT was divided into (i) fog and (ii) cloud. In the fog environment, the storage and task processing are performed locally through the fog nodes. On the other hand, the cloud environment is a remote server with more considerable computing resources, which store historical data and processes high-technological-cost tasks. In short, the main goal of HOsT is to ensure efficiency in communication for data transfer among devices in the same environment while providing efficiency in the restricted computing resources of the objects without any processing or scalability problems. To enable a better understanding of HOsT, Section 3.1 presents the chosen fog environment. Then, Section 3.2 presents the employed communication mechanism. Lastly, practical applications of HOsT are presented in Section 3.3.

### 3.1. Fog-Computing Environment

**Figure 2:**
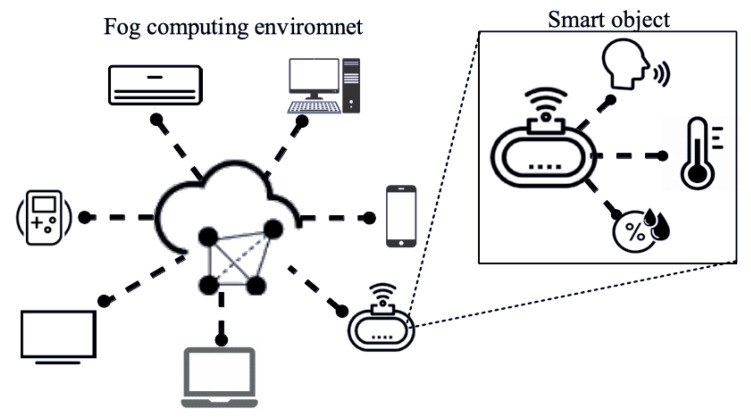
Overview of the fog environment and its respective nodes.

On HOsT, smart objects are called fog nodes and are responsible for collecting, processing, and disseminating data in the residential environment, as shown in Figure 2. In the fog-computing environment, it is assumed that each smart object is equipped with storage capacity, processing power, and a wireless communication interface. Moreover, these objects are on the network edge and are responsible for promoting communication between the users and the cloud server. The fog nodes aim to create a computing environment capable of making decisions in an area that needs to send data with better performance than that of data in a cloud environment. It should be noted that the scope of this study is not to propose models and applications for decision making, but rather to propose a solution to them.

The fog nodes are distributed in the environment and operate both as information producers and consumers. Each smart device possesses local and global knowledge of the environment. To this purpose, HOsT adopts a virtual fog map based on the work of [25]. To generate the map, the fog node shares the information through the request–reply model of Pub/Sub. Sharing is performed via a communication mechanism implemented on HOsT—presented below.

### 3.2. Communication Mechanism

The proposed solution was designed based on MQTT (message queue telemetry transport) [13] in the fog environment. This protocol contains basic concepts that are composed of: (i) publisher/subscriber—devices that send and receive messages, respectively, in an uncoupled and asynchronous way between devices [13]; (ii) topics and subscriptions—topics can be listed as the subject of the message sent by the publisher, while subscribers subscribe to topics so that they receive messages only referring to those topics; (iii) quality of service (QoS)—consists of guaranteeing message delivery and is divided into QoS0 (once at most), QoS1 (at least once), and QoS2 (exactly once); (iv) message retention—messages remain in the broker so that future subscribers of the topic in question will receive the messages [26].

It should be noted that, for the operation of this protocol, two components are required in its architecture: (i) clients, which function like any object that acts as a publisher or subscriber; and (ii) a broker, which acts as a distribution control mechanism of messages sent by clients, because it collects messages sent by the publisher and redirects them to subscribers interested in the message topics [26].

**Figure 3:**
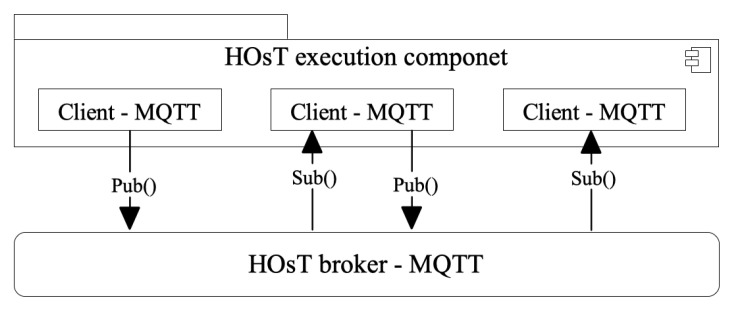
Pub/Sub communication protocol.

The choice for selecting MQTT was a result of its minimal usage of bandwidth, low imposition of resources upon the equipment (which can still reliably transmit), guaranteed delivery of messages sent through it, and decoupling among the components that constitute the environment. These are the characteristics featured in this research. Meanwhile, the publisher and subscriber are devices that send and receive messages, respectively, in a decoupled and asynchronous manner among the devices [13]. Therefore, HOsT has three components working on the MQTT process, Figure 3: (i) the publisher—the component that sends data through an event; (ii) the event—the component that generates a notification; and (iii) the subscriber—the component that receives data of interest about an event [22].

On HOsT, the decoupling among the objects is a dissociation of space, time, and synchronization. As for space, there is no need for the nodes to know about the number of publishers and subscribers contained in the fog, since the activity of each equipment that uses MQTT is independent. Moreover, there is no need for the publishers and subscribers to be active at the same time for a a message exchange to occur between them. In this context, by publishing an event, even if the subscriber is inactive at the moment the notification is generated, it can be triggered, and there is no need for the publisher to be active for the event to be directed to its subscriber once this task falls on the MQTT broker on HOsT. In addition, HOsT offers the publishers—through the MQTT—the possibility of generating events without the need for some of them to be blocked or prevented from sending messages. The subscribers work similarly in the receipt of these events when the broker is in charge of ensuring the sending of the messages to the respective subscribers. Consequently, the described solution is simplified through the asynchronous execution of its activities, enabling concurrent tasks to be performed among all the fog nodes contained in the environment to address the problem of data heterogeneity and interoperability among the nodes.

For the management of the events, HOsT uses the broker of Eclipse Paho, in the MQTT library (https://www.eclipse.org/paho/, accessed on 27 May 2022) broker, which constitutes a component of the MQTT protocol, Figure 3. The library captures the data sent by the publishers, and it generates a notification and directs it to its respective subscribers. For this to happen, both publishers and subscribers use the commands publish and subscribe, respectively, when importing the library to communicate with the broker. Additionally, the communication provided by each smart object refers to network transport occurring over a WebSocket connection, while the protocol runs over TCP/IP.

### 3.3. Applicability

From the development of HOsT, it is possible to create various applications and models for decision making that can be employed in smart environments. This solution can be used for environmental control and monitoring applications to perform decision-making processes for elderly care in a health-smart home. The aforementioned scenario employs HOsT to collect heterogeneous data such as voice, temperature, humidity, video, and body thermometer data. From this gathered data, each Pub device would send the subject to which the data and the registered message refer to the Sub of interest. Thus, real-time evaluations would be conducted, keeping track, for example, of the user’s wellbeing.

Another application that HOsT can provide is the constant verification of shopping malls’ cooling systems. This is due to the data transmission optimization on account of the fog environment among the area temperature sensors, as shown on HOsT. To maintain a pleasant temperature for all clients, it would be possible to track in the areas of a mall where there are crowds through increased room temperature caused by people’s body temperature in these areas. In addition to that, the cooling power could be increased in such places. On the other hand, from the collected data, it is possible to save energy in the mall itself. This energy-saving process would be possible through the reduction of cooling power in places with no detection of large crowds, maintaining the local temperature.

Furthermore, considering the current sociopolitical context and the crisis caused by the novel coronavirus (COVID-19), there is a possibility to identify crowds through presence sensors or even temperature sensors. As soon as a group of people is identified, those in charge could be notified and, thereby, take the necessary precautions.

## 4. Performance Evaluation

This section presents the modeled scenario along with the generated results from the performance review of HOsT. HOsT was validated in two steps, as follows: (i) evaluation of the data dissemination in the network; and (ii) evaluation of a fog-computing environment, comparing it with a baseline that sends the information directly to the cloud. Through these evaluations, it was possible to determine the functioning of HOsT and its advantages in a fog environment. Next, the modeled scenario will be presented, along with the selected parameters and the measures used to generate the results.

### 4.1. Scenario

To assess the proposal, the docker container was employed to simulate Pub/Sub devices, where such devices would represent the fog nodes on HOsT. The programming language used was Python 3.6 with the paho-mqtt (https://pypi.org/project/paho-mqtt/, accessed on 27 May 2022) library operating as a broker. The following metrics were used to assess infrastructure computing resources: (i) response time for sending a subject; (ii) data rate between device and infrastructure; and (iii) delivery rate, which represents the rate per second of topics in the infrastructure. To obtain the measures, the psutils (https://pypi.org/project/psutil/, accessed on 27 May 2022) library on Python was employed, which collects information from the processes and the operating system. In addition, to assess HOsT with a baseline that sends the information directly to the cloud, the following measures were used: (i) energy consumption, that represents the total energy consumed by the system; (ii) response time, that represents the time to complete the execution of a given task; and (iii) MIPS, that represents the number of instruction executions completed by the system per second.

For the generated results, a variation in the number of fog nodes (5, 10, 20, and 30) and in the number of subjects (8, 13, 21, and 34) was employed to investigate the impact on HOsT, as presented in Table 2. In this case, the publisher fog nodes simultaneously produced their data while subscribers received them through the broker. In addition, the payload of the subjects (100 kB, 200 kB, 400 kB, 800 kB, and 1600 kB) was varied by instantiating a broker, and the data were normalized for the response time (ms) and the data traffic (kB/s). Each experiment was performed 32 times with a trust gap of 95% using the t-Student distribution, as presented in the following subsections.

### 4.2. The Impact of HOsT Infrastructure Computing Resources

**Figure 4:**
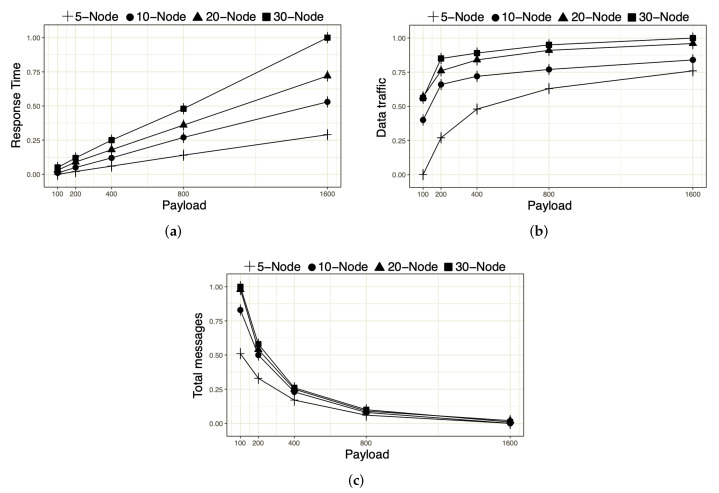
The impact of response time, data rate and message delivery. (**a**) Response Time; (**b**) Data traffic; (**c**) Message delivery rate.

HOsT performance was evaluated according to the increase in the number of fog nodes as a function of the payload sent by them. As the amount of payloads increases, growth is observed in message sending and response time, as shown in Figure 4a. This is related to the broker, which requires a longer return time due to the capture and sending of messages.

With regard to the data rate, as analyzed in Figure 4b, the stability in growth after the 400 kB payload is noted. This fact ratifies the possibility of increasing the number of devices without overloading HOsT’s infrastructure, thus avoiding the generation of an obstacle to data traffic that occurs during the process. It is noteworthy that, with the increase in the number of devices depending on the payload, the message delivery rate decreases, as observed in Figure 4c. This result comes from the speed and amount of data that the broker can receive and pass on to the remaining subscribers on the subjects in question. However, even if the delivery rate decreases as the payload increases, it is clear that the quantity of nodes does not significantly impact the result, thus achieving a stable delivery rate. Therefore, in addition to being efficient regarding the data rate, HOsT also provides good results for the message response and delivery time with payload increases. Consequently, across the impact of all metrics analyzed, it will not be challenging to predict the cost of the metrics, and it will become easier to create a fog environment with a view to the engagement analysis of the resources which will be needed for the operation to occur without problems.

### 4.3. The Impact of HOsT When Compared with a Baseline

**Figure 5:**
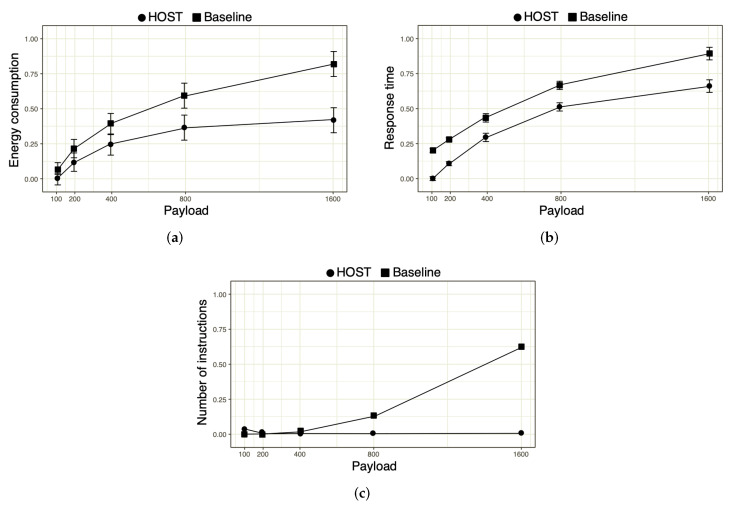
The impact of energy consumption, response time, and the number of instructions. (**a**) Energy; (**b**) Response time; (**c**) MIPS.

Figure 5a shows the result of the energy consumption by the fogs nodes as a function of the data sent by them. Note that, with 1600 kB of payload, the energy consumption spent by HOsT was significantly lower when compared with the baseline. The same behavior happens when processing 400 kB and 800 kB of data. When processed at 100 kB, HOsT is still better than the traditional approach. This is because there was no need to transmit data to process 100 kB of data. In other words, the processing might have been performed locally. Therefore, regardless of the amount of data processed, the proposed solution has a superior performance in energy efficiency when compared with the traditional approach. This approach is related to cloud systems, which have a high instance of data and, consequently, a higher delay.

After analyzing the energy consumption of the fog-computing environment, the response time taken to complete the data execution was investigated, as shown in Figure 5b. It is observed that, regardless of the scenario, as the amount of processed data increases, HOsT has faster responses than the traditional approach. On average, the processing of the proposed solution was 20% faster when compared with the traditional approach. A worst-case scenario occurs when processing 1600 kB of data and the best-case scenario occurs when processing 100 kB of data. This gain in response time makes sense since a large part of the data was processed locally.

Figure 5c shows the number of instructions executed in the fog-computing environment and the cloud environment as a function of the processed data. Except for 100 kB of processed data, it is essential to note that HOsT has a constant number of instructions executed as the amount of processed data increases. The most accentuated growth in the baseline is related to the direct transfer of information to the remote server. That is, it does not use the fog-computing environment to process data locally.

Evidently, after analyzing Figure 5, it is possible to infer from the analyzed metrics that resource consumption will not only be better than the attractive approach but also related to the easy prediction of resource consumption given by HOsT.

## 5. Conclusions

This research addresses the problem of data heterogeneity and interoperability of smart objects in the residential context. As a result of this work, we proposed HOsT, an infrastructure that abstracts the communication details of smart objects based on the Pub/Sub module. The performance evaluation presents evidence of the efficiency in the devices’ computing resources and the communication infrastructure of HOsT. The results have shown that HOsT accommodates scalability—changes the number of devices acting simultaneously—and has been demonstrated to be able to operate with different types of devices and data.

The main findings are related to easy operating and computing the cost forecasting of the fog environment. This is due to the result projection stability and patterns regarding response time performance, data rate, and message delivery.

In future works, the research of this paper will be expanded to implement HOsT in different contexts, focusing on smart environments. In addition, in the context of data security, the expansion of this work is of the utmost importance since the data captured by the residential fog environment has a private nature and should only be shared with the user’s authorization.

## Figures and Tables

**Table 1:** Comparison of this work with the related works.

Related Work	Pub/Sub	Fog Computing	Scalability
Kang et al. [18]	√		
de Sousa et al. [19]	√		√
Jutadhamakorn et al. [17]	√		√
Rocha Filho et al. [21]	√	√	√
Kumar [16]		√	
Dastjerdi et al. [23]		√	√
HOsT	√	√	√

**Table 2:** Set of parameters employed to generate the results.

Parameters	Standard Value
Number of Topics	8, 13, 21 and 34
Number of fog nodes	5, 10, 20 and 30
Payload	100 kB, 200 kB, 400 kB, 800 kB and 1600 kB
Broker	1
Protocol	Eclipse Paho MQTT
Number of replications	32
Confidence interval	95%

## Data Availability

Not applicable.
